# *Porphyromonas gingivalis*: a potential trigger of neurodegenerative disease

**DOI:** 10.3389/fimmu.2025.1482033

**Published:** 2025-02-14

**Authors:** Ziyan Huang, Miao Hao, Naixu Shi, Xinyu Wang, Lin Yuan, Haotian Yuan, Xiaofeng Wang

**Affiliations:** ^1^ Department of Stomatology, China-Japan Union Hospital of Jilin University, Changchun, Jilin, China; ^2^ Scientific Research Center, China-Japan Union Hospital of Jilin University, Changchun, Jilin, China

**Keywords:** *Porphyromonas gingivalis*, neurodegenerative diseases, periodontitis, Alzheimer’s disease, Parkinson’s disease, neuroinflammation

## Abstract

*Porphyromonas gingivalis* (*P. gingivalis*) is a gram-negative bacterium and the main causative agent of periodontitis, a disease closely associated with the development of periodontal disease. The progression of periodontitis, a chronic infectious disease, is intricately linked to the inflammatory immune response. Inflammatory cytokines act on periodontal tissues via immunomodulation, resulting in the destruction of the periodontal tissue. Recent studies have established connections between periodontitis and various systemic diseases, including cardiovascular diseases, tumors, and neurodegenerative diseases. Neurodegenerative diseases are neurological disorders caused by immune system dysfunction, including Alzheimer’s and Parkinson’s diseases. One of the main characteristics of neurodegenerative diseases is an impaired inflammatory response, which mediates neuroinflammation through microglial activation. Some studies have shown an association between periodontitis and neurodegenerative diseases, with *P. gingivalis* as the primary culprit. *P. gingivalis* can cross the blood-brain barrier (BBB) or mediate neuroinflammation and injury through a variety of pathways, including the gut-brain axis, thereby affecting neuronal growth and survival and participating in the onset and progression of neurodegenerative diseases. However, comprehensive and systematic summaries of studies on the infectious origin of neurodegenerative diseases are lacking. This article reviews and summarizes the relationship between *P. gingivalis* and neurodegenerative diseases and its possible regulatory mechanisms. This review offers new perspectives into the understanding of neurodegenerative disease development and highlights innovative approaches for investigating and developing tailored medications for treating neurodegenerative conditions, particularly from the viewpoint of their association with *P. gingivalis*.

## 1 Introduction

The aging population is leading to an annual increase in neurodegenerative diseases like Alzheimer’s disease (AD) and Parkinson’s diseases (PD), which poses a significant threat to human health. Currently, effective treatments strategies and drugs are lacking, suggesting that some key pathogenic mechanisms may remain undiscovered. Recent studies indicate that *Porphyromonas gingivalis*, the primary bacterium responsible for periodontitis, may significantly contribute to the onset and progression of neurodegenerative diseases.

Periodontitis, a common chronic inflammatory disease, affects over 700 million people worldwide and is frequently overlooked and untreated (1). *Porphyromonas gingivalis* (*P. gingivalis*) is the primary pathogenic bacterium causing periodontitis, with approximately 85.75% of subgingival plaque samples testing positive for this opportunistic bacteria (2). Typically, inflammatory factors such as C-reactive protein, IL-6 and IL-21 are significantly upregulated in serum of patients with *P. gingivalis* -induced periodontitis (3). This is a distinctive feature of Chronic Low-Grade Inflammatory Phenotype (CLIP), which often occurs during the aging process (4, 5). Studies have shown that periodontitis is an important risk factor for various health issues, including cardiovascular diseases, diabetes, and Parkinson’s disease (6, 7). Recent research found *P. gingivalis* in the brains and spinal cord of Alzheimer’s patients (8). Furthermore, study confirms that *P. gingivalis* infection enhances BBB permeability, and gingivally infected *P. gingivalis* may cause cognitive decline with periodontitis (9, 10). These findings confirm the strong link between P. gingivalis and the development of neurodegenerative diseases.

Most studies on the relationship between periodontitis, *P. gingivalis* periodontitis, and neurodegenerative diseases have focused primarily on clinical and epidemiological perspectives. However, there is little research on the specific regulatory mechanisms connecting the two, and the existing summaries are inadequate. This review aims to closely examine the association and potential mechanisms linking *P. gingivalis* and neurodegenerative diseases, including the roles of bacteria, inflammation, and immune system responses. Through this review, we hope to propose some innovative concepts to clarify the origins of neurodegenerative diseases. At the same time, we also hope to offer fresh ideas for preventing and treating these diseases.

### 1.1 Porphyromonas gingivalis

*P. gingivalis* colonizes the oral epithelium and forms part of the plaque beneath the gums. It can alter the symbiotic composition of bacteria in the oral cavity, leading to ecological dysbiosis. The production of *P. gingivalis* biofilms is associated with the formation of bacterial plaques in gingival tissue, which further exacerbates gingival damage by other oral bacteria (11, 12).

### 1.2 Mechanisms of *P. gingivalis* involvement in neurodegenerative diseases

*P. gingivalis* possesses several unique properties and virulence factors that affect the host (2). One notable characteristic is the shedding of outer membrane vesicles (OMVs). These vesicles contain virulence factors, particularly gingival proteases and lipopolysaccharide (LPS) (13, 14). Gingipain triggers an immune escape response by modulating inflammatory mediators and suppressing immune cell activity, including the activities of the lysine-gingipain (Kgp) and arginine-gingipain (Rgp). *P. gingivalis* also produces *P. gingivalis* lipopolysaccharide (P.g-LPS), which activates the natural host immune response (15, 16). Most strains of *P. gingivalis* are covered by a capsule that protects the bacteria from attack and host complement killing (17). Some studies have shown that encapsulated strains are more virulent in a mouse model of infection (18, 19). Therefore, these toxins make *P. gingivalis* highly pathogenic, enabling its components to enter the brain through various pathways. This entry triggers pathological reactions and contributes to the development of neurodegenerative diseases.

**Figure 1:**
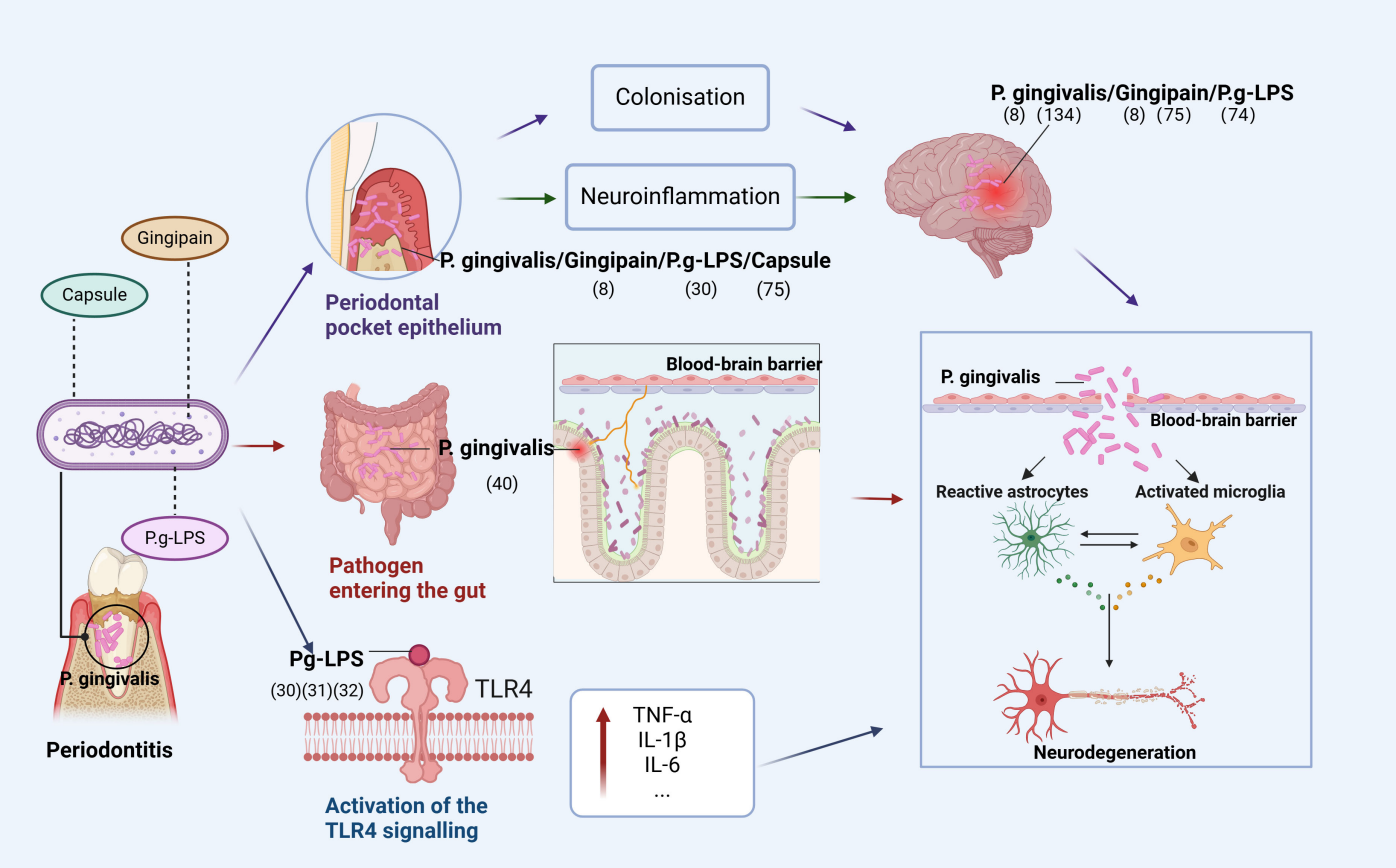
Pathways of *P. gingivalis* to the brain. The figure depicts that *P. gingivalis* virulence factor P.g-LPS can enter the brain through three pathways: (1) enters the epithelium of periodontal pockets to cause local inflammation and indirectly promotes neuroinflammation or directly colonize the brain; (2) disrupts the homeostatic balance of intestinal flora and enters the brain through the gut-brain axis to cause neuroinflammation; (3) activates the TLR4 signaling-inducing OS, leading to mitochondrial dysfunction and neuroinflammation (Figure was created with BioRender.com. Huang, Z. (2025) https://BioRender.com/a16s851).

Based on current research, we conclude that oral infections caused by *P. gingivalis* can affect the brain in three ways (**Figure 1**). First, *P. gingivalis* causes local chronic inflammation and disrupts central nervous system (CNS) homeostasis through the blood-brain barrier, indirectly promoting neuroinflammation. The association of high loads of *P. gingivalis* with increased serum TNF-α levels suggests that *P. gingivalis* not only triggers the development of local inflammatory periodontitis but also leads to elevated serum levels of pro-inflammatory cytokines (20). In addition, prolonged exposure to harmful substances disrupts and increases blood-brain barrier permeability, allowing peripheral pro-inflammatory cytokines to enter the vagus nerve (21).

Second, *P. gingivalis* enters the intestine through the mouth and can disrupt the intestinal flora, leading to an inflammatory response transmitted to the brain through the gut-brain axis (GBA). Dysregulation of the gut microbiota is strongly associated with the development of neurodegenerative diseases through the regulation of the GBA, and *P. gingivalis* can alter the ratio of T lymphocytes to inflammatory T cells in mesenteric lymph nodes and increase inflammatory cytokines, disrupting the gut microbiota (22–24). Gut microbiota can alter immune cells and stimulate the production of pro-inflammatory cytokines (25–27), with immune cells and inflammatory mediators subsequently entering the brain (28).

Finally, TLR4 signaling activated by *P. gingivalis* induces OS, leading to mitochondrial dysfunction and neuroinflammation. In both cases, it is involved in the progression of degenerative diseases due to neuroinflammation. A recent study showed that Pg-LPS acts via TLR4. Furthermore, administering Pg-LPS triggers TLR4 signals and elevates markers of dementia and neuroinflammation that have been linked to AD (29). Additional research has revealed that neuroinflammation caused by Pg-LPS is facilitated by the activation of the TLR4 signaling route (30, 31). Research indicates that neurons are capable of expressing both TLR2 and TLR4, implying that these receptors are crucial for neuroinflammatory responses (32, 33).

## 2 Neurodegenerative diseases

Neurodegenerative diseases are disorders that impact the central nervous system, such as AD, PD, multiple sclerosis (MS), amyotrophic lateral sclerosis (ALS), and Huntington’s disease (HD). These diseases are commonly associated with aging, and their occurrence rises as people get older (34). Immune responses from activated neuroglial cells is crucial in neurodegenerative diseases, particularly in the most prevalent forms: AD and PD (35). Neurodegenerative diseases target various areas of the brain, resulting in different symptoms and causes. Despite these differences, neurodegenerative diseases share common features and involve similar cellular and molecular processes.

### 2.1 Pathogenetic mechanisms of neurodegenerative diseases

An imbalance in the inflammatory response is a common issue in neurodegenerative diseases that leads to neuroinflammation in various parts of the brain, worsening the overall condition (36). The released mediators impact microglia and astrocytes, potentially harming neurons and the central nervous system (36–38). For instance, pyroptotic cell of microglia can lead to neuroinflammation, triggering various neurodegenerative diseases (39). Similarly, damage to astrocytes can lead to specific neuroinflammatory markers, resulting in further complications (40). Another notable feature of neurodegenerative diseases is oxidative stress. In the brains of patients with neurodegenerative diseases, elevated levels of reactive oxygen species are often seen, suggesting that oxidative damage might make the disease worse (41, 42).

### 2.2 Neuroinflammation and brain immune cells

Inflammation is the response of cells and tissues to injury, trauma, or infection. Research shows that there is a significant link between the brain and the immune system. Inflammation occurring in the brain is called neuroinflammation (43). Neuroinflammation is an early feature of neurodegenerative diseases. It can activate the immune system, leading to the release of pro-inflammatory cytokines, increased oxidative stress (OS), and abnormal protein deposition, all of which may harm neurons directly or indirectly (44–46). For instance, while neuroinflammation can help remove deposited Aβ, it can also generate cytotoxic substances that worsen Aβ deposition and contribute to neurodegenerative damage (47). Recent studies suggest that neuroinflammation might be a result of periodontitis (8). The immune cells in the brain mainly consist of microglia and astrocytes. Chronic inflammation in the body triggers the activation of microglia and astrocytes in the brain. For instance, when the Toll-like receptors (TLRs) in these cells get activated, they produce various pro-inflammatory cytokines, leading to neuroinflammation that causes damage or death to neurons (48–50).

#### 2.2.1 Microglia and neuroinflammation

Microglia are innate immune cells that participate in synaptic remodeling and defense functions, involved in homeostasis and host defense against pathogens and CNS diseases (51). Under normal conditions, microglia are highly branched and perform their sensory functions (52). With aging and chronic stress, microglia activation exhibits dystrophic morphology and an exaggerated inflammatory response (53). Although neuroinflammation is a neuroprotective mechanism, it induces neurotoxicity and is associated with neurodegeneration (51). In neurodegenerative diseases, microglia can migrate the site of injury, produce cytokines such as tumor necrosis factor (TNF)-α, interleukin (IL)-1β, and accumulate pathogenic proteins that mediate neuroinflammation (49).

#### 2.2.2 Astrocytes and neuroinflammation

Astrocytes, the most abundant glial cells in the CNS, perform various functions in healthy neural tissues, including the regulation of blood flow and extracellular fluid, ionic and transmitter balance, energy supply, and synaptic function (54). Astrocytes are activated in response to pathological stimulation. During neuroinflammation, astrocytes enhance the activity of the IL-17 receptor, a crucial inflammatory agent released by effector T lymphocytes (55). The binding of IL-17 to various transmembrane receptors results in the activation of NF-κB-activating factor 1 (Act1) and the formation of signaling complexes, leading to the synthesis of pro-inflammatory cytokines, chemokines, and metalloproteinases (50).

The role of astrocytes in neuroinflammation is twofold. Astrocytes can reduce inflammation by releasing anti-inflammatory factors and promoting the production of neuroprotective factors. Also, they can exacerbate inflammation and nerve damage by releasing pro-inflammatory and neurotoxic molecules. Thus, the role of astrocytes in neuroinflammation is complex and encompasses pro-inflammatory, inhibitory, and pro-neuroprotective effects.

## 3 *P. gingivalis* and neurodegenerative diseases

*P. gingivalis* has significant implications in the pathophysiology of neurodegenerative diseases because its virulence factors can enter the brain through three pathways, causing OS and neuroinflammation. First, toxic proteases produced by *P. gingivalis*, such as gingipains, may directly damage neurons in the brain, leading to the activation of microglia and astrocytes, thereby inducing neuroinflammation (56). Second, *P. gingivalis* infection may enter the brain through blood circulation, causing local and systemic inflammatory responses that may form positive feedback loops with oxidative stress and neurodegenerative changes (57). In addition, *P. gingivalis* infection may further exacerbate neuroinflammation by promoting the accumulation of misfolded proteins, such as Aβ and tau proteins (8, 58). The interaction between the abnormal accumulation of these proteins and neuroinflammation may lead to the onset and development of neurodegenerative events such as AD and PD.

### 3.1 *P. gingivalis* and AD

AD is characterized by the progressive cognitive decline resulting from synapse degeneration and neuronal death. It is an irreversible chronic degenerative neurological disease and the most common neurodegenerative disease leading to cognitive impairment (59, 60). AD is associated with cognitive dysfunction, Aβ plaques and neurofibrillary tangles (NFTs) formed by hyperphosphorylated tau are the two hallmark pathological features of AD (61, 62). Aβ plaques are recognized by the brain as foreign bodies, triggering inflammatory and immune responses through activation of microglia and cytokine release, ultimately leading to cell death and neurodegeneration (63). And phosphorylated tau proteins contribute to AD by causing microtubule rupture, synaptic loss, and, ultimately, cognitive dysfunction (64, 65). Moreover, both Aβ and tau aggregate impair synaptic plasticity and lead to neuronal cell death (66).

Firstly, *P. gingivalis* could induce neuroinflammation and neurodegeneration via OS. Le Sage et al. found that at the cellular level, P.g-LPS induces oxidative stress by increasing intracellular ROS production and altering the expression of genes encoding the oxidoreductases NOX2, NOX4, iNOS, and catalase (67). Accumulation of ROS, reduced MMP expression, and increased 4-HNE protein expression in neuroblastoma cells due to P.g-LPS underscore its significance in the pathogenesis of AD (29). Furthermore, LPS from *P. gingivalis* increases OS in periodontal ligament fibroblasts and brain endothelial cells (68, 69).

In addition to inducing neuroinflammation through OS, *P. gingivalis* can directly cause neuroinflammation. Animal studies have shown that P.g-LPS-induced neuroinflammation leads to cognitive impairment in C57BL/6 mice and that P.g-LPS significantly activates astrocytes and microglia and upregulates the TLR4/NF-κB signaling pathway (70). Hu et al. found that periodontitis caused by P.g-LPS exacerbates neuroinflammation by stimulating TLR4 and the NF-κB signaling pathway, which have been linked to learning and memory deficits in Sprague-Dawley rats (71). *P. gingivalis* OMV, which carries high levels of gingipain, may play a significant role in AD (72). Research have demonstrated that LPS derived from *P. gingivalis* OMV activates glial cells and induces brain inflammation. It is also linked to the expression of AD markers, such as Aβ and NFTs (73). In addition, the capsules of *P. gingivalis* play a central role in chronic inflammatory responses and cognitive deficits caused by short-term oral infections. More toxic capsules are likely to induce AD-like pathology and accelerate the pathogenic process (74).

At the same time, the pathogenic factors of *P. gingivalis* can also lead to the development of pathological features associated with AD, such as influencing Aβ accumulation and tau protein function. Animal studies corroborate that oral *P. gingivalis* infection in mice leads to brain colonization and enhances the generation of the amyloid plaque element Aβ1-42 (8). Similarly, Ryra et al. reported, using a rat model, that P.g-LPS induces an increase in serum levels of Aβ peptide (75). Tang et al. demonstrated that rats infected with *P. gingivalis* exhibit robust tau phosphorylation at the Thr181 and Thr231 loci linked to AD, and these loci are abundant in activated astrocytes (76).

At the cellular level, it has been established that prolonged contact with P.g-LPS led to the buildup of Aβ in the brains of mice of middle age. The exposure further led to the peripheral build-up of Aβ in inflammatory monocytes and macrophages (58). Gingipains, another toxic product of *P. gingivalis*, are associated with tau phosphorylation and tau cleavage (77, 78). Dominy et al. suggested that the origin of tau in the brains of patients with AD could stem from the transneuronal spread of *P. gingivalis*, Gingipain may also play a role in the adaptive elevation of tau protein synthesis in patients with AD (8).

In summary, *P. gingivalis* has been linked to the pathogenesis of AD through multiple mechanisms, including neuroinflammation, oxidative stress, Aβ accumulation, and interference with tau protein function, as summarized in **Table 1**. These findings suggest that *P. gingivalis* may contribute to the development of AD through different pathways, whether at the cellular or animal, level, thus providing potential targets for future therapeutic strategies.

**Table 1:** P*. gingivalis* affects the development of AD.

Mechanism/Target	Type of study	Virulence	Published time	Reference
Direct infection Direct infection of the brain, leading to nerve cell damage	Clinical	Gingipain	2019	(8)
	Animal experimental	*P.gingivalis*/Gingipain	2023	(10)
	Epidemiological	*P.gingivalis*	2017	(79)
	Animal experimental	*P.gingivalis*	2023	(9)
Inflammation-mediated *P. gingivalis* activates inflammatory pathways, indirectly leading to neurodegeneration	Clinical	Gingipain	2019	(8)
	Epidemiological	*P.gingivalis*	2017	(79)
	Epidemiological	*P.gingivalis*	2008	(80)
	Animal experimental	*P.gingivalis*/Gingipain	2018	(81)
	Cellular level	LPS	2013	(82)
Aβ production *P. gingivalis* affects Aβ production and clearance, leading to Aβ deposition	Animal experimental	LPS	2013	(82)
	Epidemiological	*P.gingivalis*	2008	(80)
	Cellular level	*P.gingivalis*	2020	(74)
Tau phosphorrylation *P. gingivalis* affects the phosphorylation state of tau protein and promotes the formation of neuro-fibrillary tangles	Cellular level	*P.gingivalis*/Gingipain	2018	(81)
	Animal experimental	*P.gingivalis*	2023	(83)
	Cellular level	*P.gingivalis*	2020	(74)

### 3.2 *P. gingivalis* and PD

PD is the second most prevalent neurodegenerative disorder that results from the death of dopaminergic nerve cells in the substantia nigra pars compacta (SNPC) (84, 85). It leads to movement disorders such as tremors, bradykinesia, and cognitive impairment (86). Pathologically, PD involves alpha-synuclein (α-Syn) misfolding, neuroinflammation, and mitochondrial dysfunction (87, 88). In recent years, periodontitis, a common slow inflammatory disease, has been associated with the risk of PD (89, 90). Most previous associations between the two diseases have been based on PD-induced dyskinesias, which may lead to the progression of periodontal disease (90). And *P. gingivalis* is a major periodontal pathogen that induces intestinal dysbiosis (91, 92).

Our previous study employed bioconfidence data mining to demonstrate that periodontitis is a high-risk causative factor of PD, and our results suggest that *P. gingivalis*, the main causative agent of periodontitis, can contribute to the development of PD (93). Previous studies have also detected *P. gingivalis* major virulence factors such as gingipain R1 and P.g-LPS in the blood of PD patients (94, 95). A recent study suggests that gingipains from *P.gingivalis* may accumulate in the SNPC of the human brain (96). An animal study confirmed that *P. gingivalis* reduces dopaminergic neurons in SNPC of mice with the leucine-rich repeat kinase 2 (LRRK2) R144G mutation, which is associated with late-onset PD (97, 98).

*P. gingivalis* may affect the onset and development of PD primarily through two mechanisms: OS and neuroinflammation. This mechanism was confirmed in various studies on animal models, including a report by La Vitola et al., which found that LPS from *Escherichia coli* (*E. coli*) induces neuroinflammation and enhances α-Syn toxicity along with cognitive impairment (99). Similarly, a previous study showed that P.g-LPS hindered spatial learning and memory in the Morris Water Maze (MWM) test, whereas the effects of the two LPS types were not significantly different (70). The findings imply that P.g-LPS, notwithstanding its structural variances, might possess a mechanism akin to *Escherichia coli* LPS that leads to the intensification of harmful impacts of α-Syn and cognitive deficits.

There is currently no direct evidence that *P. gingivalis* contributes to the development of PD via oxidative stress. However, studies indicate that P.g-LPS induces oxidative stress, leading to mitochondrial dysfunction and neuroinflammation in SH-SY5Y cells (29). In addition, a positron emission tomography (PET) study of patients with PD have shown that a widespread presence of activated microglia (100). Interestingly, this response does not correlate with clinical severity, suggesting it may occur early in the disease. The mechanism by which microglia are involved in PD may be similar to that seen in AD (51). Microglia internalize and degrade the proteinα-Syn. If this process fails, extracellular α-Syn accumulates, similar to Aβ (101). Microglia gather around α-Syn deposits and display pro-inflammatory properties base on receptors that also bind Aβ (102, 103). Therefore, we hypothesized that *P. gingivalis* may contribute to PD through oxidative stress and neuroinflammation.

### 3.3 *P. gingivalis* and MS

MS is a progressive disorder of the central nervous system, characterized by invasion by immune cells, detachment of myelin sheaths, growth of reactive glial cells, and damage to neural axons. This sequence results in sensory, motor, and cognitive impairments (104). Although the exact cause of MS recurrence remains unclear, it is believed to stem from an autoimmune inflammatory condition in which environmental and genetic elements trigger CNS antigens, such as myelin basic protein, to be addressed by the immune system (105).

The correlation between MS and *P. gingivalis* is currently being investigated, although there are no published articles describing the role of *P. gingivalis* in the pathogenesis of MS. As one of the most prevalent neurodegenerative diseases, MS is also associated with neuroinflammation. Lucchinetti et al. showed that acute MS leads to astrocyte and microglial activation and occasionally to oligodendrocyte apoptosis (106).

Some relevant experiments have sought to demonstrate this link, including a report by Moreno et al. aimed to investigate whether systemic inflammatory stimuli exacerbate axonal damage. Through the use of rat models of autoimmune encephalomyelitis, the researchers showed that microglia activation leads to an increase in carbon monoxide synthase, IL-1β, and axonal damage (107). The findings confirmed a relationship between peripheral inflammation and neurodegeneration in a rodent model (91). In another study, Polak et al. engineered an MS mouse model by infecting it with *P. gingivalis*. Their experiment showed that tail weakness and paralysis in the extremities developed while exacerbating MS pathology and increasing lymphocyte proliferation (108). These findings suggest that *P. gingivalis* can contribute to the exacerbation of MS pathology by causing an inflammatory response.

### 3.4 *P. gingivalis* and ALS

ALS is the most common motor neuron (MN) disease, with an average age of onset of 50-65 years (109). Neuroinflammatory processes induced by microglia and astrocytes appear to play an important role in ALS pathology. Reactive astrogliosis occurs under pathological conditions such as ALS, shifting these cells from a ‘neuroprotective’ to a ‘neurodegenerative’ role (110).

In ALS, astrocyte activation is associated with motor neuron degeneration, which promotes inflammation and OS. In the early stages of disease, astrocytes provide neuroprotection. As the disease progresses, activated astrocytes promote a neurotoxic environment. This occurs either through microglial activation processes or through compounds released by motor neuron, ultimately resulting in motor neuron death (111). Microglia also play a key role in ALS, with M2 microglia being neuroprotective and M1 microglia being toxic. Studies in animal indicate that as the ALS progresses in fALS mice, the number of ‘neuroprotective’ M2 microglia increases. However, at later stage, these microglia transition to ‘neurotoxic’ M1 microglia (112–115). Inflammatory cytokines released by astrocytes and microglia may promote glutamatergic excitotoxicity, thereby linking neuroinflammation to excitotoxic cell death. When critical thresholds are reached, reactive astrocytes and microglia may trigger irreversible pathological processes that subsequently lead to non-cell-autonomous death of motor neurons in ALS patients (116).

Despite the absence of P. g-LPS, research has shown that microglia in ALS models can be stimulated by various LPS sources. This triggers a transition from protective to pro-inflammatory conditions, resulting in the production of IL-12, TNFα, NO, superoxide anion, and peroxynitrite. This process causes motor neuron deterioration and exacerbates the disease in mice models of ALS. Additionally, these molecules enhance the interaction between extracellular glutamate and its receptor on motor neurons, causing increased calcium influx into the cells and triggering cellular death (117–119). In ALS, the motor regions of the CNS may be affected by neuroinflammation and OS, evidenced by the activation of reactive astrocytes and microglia, moderate invasion of peripheral immune cells, and increased levels of inflammatory mediators (120). Animal models primarily show unusual growth of astrocytes and the presence of inflammatory indicators such as cyclooxygenase-2, inducible NOS, and neuronal NOS (121). Clinical studies have found that astrocytes in the spinal cords of patients with ALS are cytotoxic to MN in culture (122). Cytokines, such as G-CSF, IL2, IL15, IL17, MCP-1 MIP1α, TNFα, and VEGF found in the cerebrospinal fluid of individuals with ALS were found to be unusually elevated (123). Furthermore, patients with ALS exhibit increased IL-6 levels in exosomes derived from astrocytes, indicating a potential role of CNS-derived exosomes in uncovering neuroinflammation in patients with ALS and a direct relationship with the rate of disease progression (124).

Moreover, in ALS, OS may play a role in the deterioration of neuromuscular junctions. Mouse models show enhanced sensitivity of nerve endings to ROS, which may lead to the degeneration of presynapses at neuromuscular junctions. Concurrently, excessive activation of excitatory amino acids leads to the irregular release of acetylcholinesterase, diminishing acetylcholine levels in the synaptic gap and potentially resulting in diminished muscle strength in patients with ALS. These initial malfunctions, coupled with diminished inflammation and nutritional aid, potentially culminate in neurodegenerative disorders (125).

Although no direct evidence exists that *P. gingivalis* is involved in the pathogenesis of ALS, we can conclude that neuroinflammation and OS are intertwined mechanisms involved in the pathophysiology of ALS. Although further research is needed to determine whether *P. gingivalis* influences ALS through mechanisms of neuroinflammation and OS, *P. gingivalis* can trigger neuroinflammation and OS, and we, therefore, hypothesize that it is involved in the development of ALS through neuroinflammation and OS.

### 3.5 *P. gingivalis* influences neuroinflammation through the gut-brain axis

Apart from the associations between *P. gingivalis* and AD, PD, and MS, which has been summarized above, no studies on the effects of *P. gingivali*s on other neurodegenerative diseases, such as ALS, have been reported. However, the unifying clinical feature or disease phenotype of these disorders is neuroinflammation. *P. gingivalis* not only induces neuroinflammation directly but also mediates neuroinflammation through the oral-intestinal-brain axis.

The oral cavity is the starting site of the digestive tract, and humans ingest approximately 1.5 × 10^12^ oral bacteria daily from swallowed saliva (126), and *P. gingivalis* of oral origin can induce dysbiosis of the intestinal flora (127, 128). For example, a clinical study found altered gut microbiota in patients with periodontitis (129). Furthermore, in a previous study, we found that periodontitis-associated periodontal pathogens disrupt the gut microbiota, exacerbate the systemic immune response, and worsen colitis (130). According to Wang et al., oral microbes can have an impact on the gut, and *P. gingivalis* was detected in the feces of patients with colorectal cancer (131).

All these studies suggest that oral pathogenic bacteria can affect the central nervous system via the Gut-brain axis, suggesting the existence of an oral-intestinal-brain axis. A previous study also defined the presence of the Oral-gut-brain axis (132). It has been shown that *P. gingivalis* affects neuroinflammation by influencing intestinal ecology by increasing the number of inflammatory T and B lymphocytes, thereby inducing neuroinflammation (133, 134). Dysbiosis of the gut microbiota reduces the production of short-chain fatty acids, which are linked to inflammatory responses (135, 136). Another study indicated that the gut microbiota affects both microglial maturation and normal function and that dysregulation of the microbiota can lead to neuroinflammation (137). Feng et al. found that oral administration of *P. gingivalis* reduces intestinal permeability and elevates IL-17a levels in the peripheral blood of R1441G mice, potentially linked to neuronal demise and neuroinflammation (97). Thus, the aforementioned studies suggest that *P. gingivalis* influences neuroinflammation via the oral-intestina-brain axis, which may be closely related to the pathogenesis of several neurodegenerative diseases.

**Figure 2:**
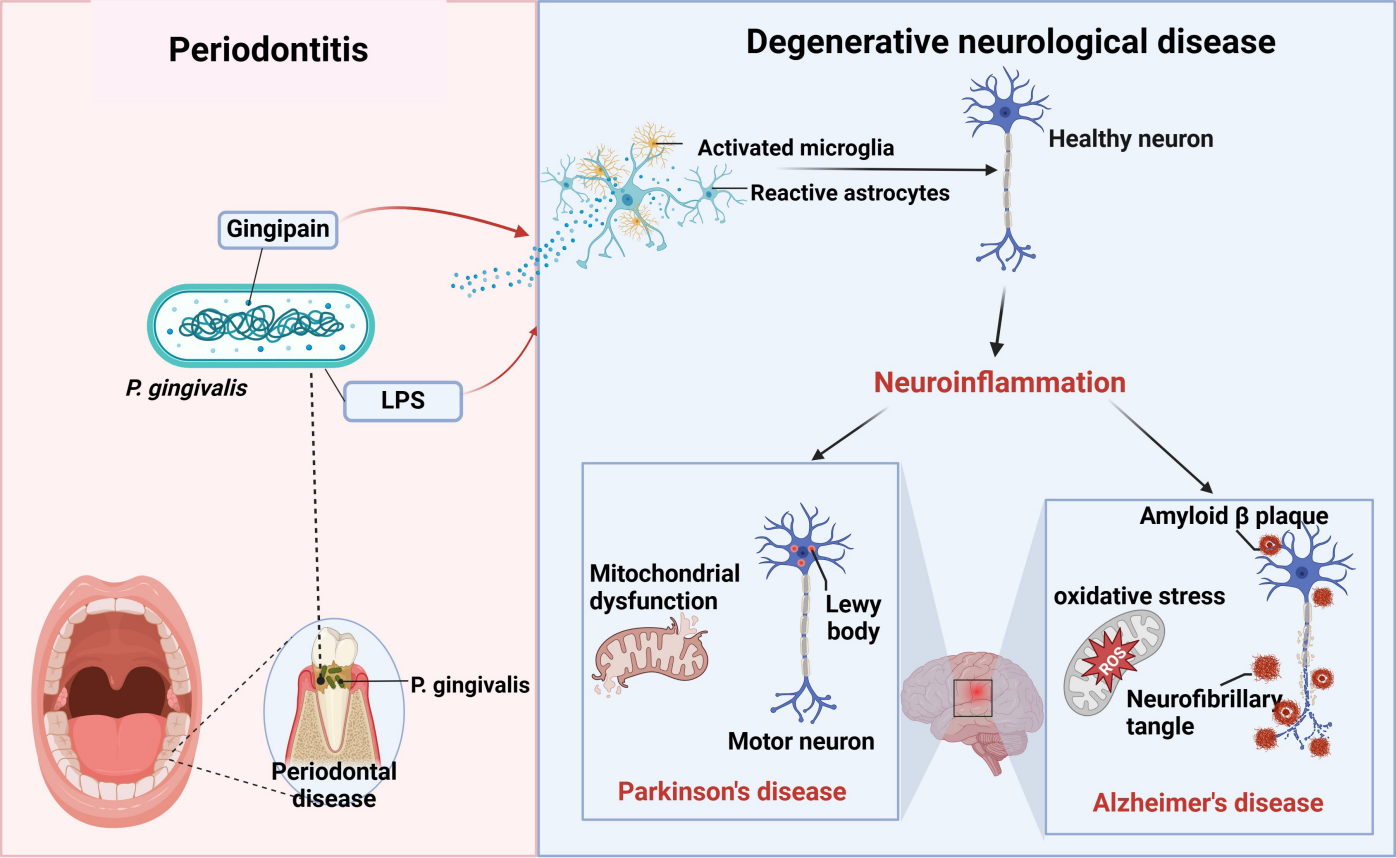
P*. gingivalis* and neurodegenerative diseases. Periodontitis triggers neuroinflammation through the production of virulence factors P.g-LPS and gingipain by *P. gingivalis*, which activate the brain’s immune cells, astrocytes and microglia, causing alterations in healthy neurons that ultimately lead to neurodegenerative diseases such as AD and PD (figure was created with BioRender.com. Huang, Z. (2025) https://BioRender.com/a16s851).

Currently, there is convincing evidence that oral *P. gingivalis* may influence neurodegenerative diseases such as Alzheimer’s and Parkinson’s diseases. Here, we summarize the relationship between the two diseases (**Figure 2**).

## 4 Discussion

*P. gingivalis* is the main causative agent of periodontitis, inducing both periodontal inflammation and systemic chronic inflammation, as well as neuroinflammation. Recent studies indicate that *P. gingivalis* is not only relevant to periodontitis, but also directly induces AD through neuroinflammation and oxidative stress, suggesting its involvement in the development of neurodegenerative diseases. This review systematically summarizes the literature regarding *P. gingivalis*’s role in the development of neurodegenerative diseases through neuroinflammation and further analyzes the underlying mechanisms involved.

Neurodegenerative diseases, such as AD, may be associated with infections. T Numerous studies have confirmed that pathogenic microorganisms, including human immunodeficiency virus (HIV) and specific herpes viruses like herpes simplex virus (HSV), are linked to neurodegenerative disorders. HIV infection can lead to AIDS-associated dementia, while HSV infection may increase the risk of AD (138, 139). Additionally, certain bacteria, such as *Streptococcus pneumoniae*, are linked to neurodegenerative diseases. For example, infections with *Streptococcus pneumoniae* can result in meningitis, potentially causing lasting neurological harm and cognitive deterioration (140). Additionally, gut microbes can influence AD through the gut-brain axis. A study found notable differences in the gut bacterial community structure between AD model mice and their age-matched wild-type counterparts. AD mice have significantly lower abundance of members of the *phyla Thick-walled Bacteria, Micrococcus wartyi, Aspergillus, and Actinobacteria*, and increased abundance of members of the phyla Synechococcus and Teneribacteria (141); these changes may lead to TNF-mediated gastrointestinal inflammation, which can increase the risk of AD (142). This suggests that shaping the composition of the gut microbiota may influence the progression of AD. These findings indicate that the mechanisms of neurodegenerative diseases are complex and, therefore, studies from the point of view of pathogenic microorganisms, especially oral flora, are of great importance.

*Porphyromonas gingivalis* impacts host’s immune function and triggers inflammatory, which may lead to various immune diseases beyond neurodegenerative disorders. Recent studies suggest a link between *P. gingivalis* and several immune disorders, including autoimmune and inflammatory conditions. In autoimmune disorders, *P. gingivalis* can trigger immune responses through molecular mimicry, leading to the production of autoantibodies. Research shows that a particular peptide (Pep19) from *P. gingivalis* heat shock protein 60 reacts significantly in the serum of individuals with autoimmune disorders. This finding suggests *P. gingivalis* may play a role in the emergence and progression of these diseases (143). Furthermore, *P. gingivalis* is associated with inflammatory disorders atherosclerosis, diabetes, and rheumatoid arthritis. Frequently associated with persistent inflammatory reactions, these conditions can worsen due to *P. gingivalis* by enhancing the inflammatory response regulation (143).

Investigating the connection between *P. gingivalis* and neuroinflammation is an emerging area of research, especially regarding neurodegenerative disorders like AD. Many studies have suggested ways in which *P. gingivalis* is connected to neuroinflammation. However, this research is still in its early stages, requiring more experimental and clinical studies to validate these links and investigate possible treatment options. In conclusion, studying the relationship between *P. gingivalis* and neurodegenerative conditions deepens our understanding of these diseases and raises public awareness of periodontitis. Improving the prevention and treatment of periodontitis may help reduce the onset and progression of neurodegenerative diseases. The intricate connection between *Porphyromonas gingivalis* and neurodegenerative diseases requires further investigation to establish a scientific foundation for their prevention and treatment.
